# Analysis of the intestinal microbial community altered during rotavirus infection in suckling mice

**DOI:** 10.1186/s12985-021-01727-5

**Published:** 2021-12-20

**Authors:** Wei Zhao, Mei Ling Yu, XiaoLi Tao, Mei Hui Cheng, Chang Cheng Liu, Yang Liu, Yong Gang Li

**Affiliations:** College of Basic Medical Sciences, Jinzhou Medical University, Jinzhou, 121200 Liaoning People’s Republic of China

**Keywords:** Diarrhea, Rotavirus, Gut microbiota, 16S rRNA gene sequencing, Suckling mice model

## Abstract

**Background:**

Rotavirus (RV) is a principal cause of diarrhea. However, there is a limited understanding regarding alteration of the gut microbial community structure and abundance during RV infection. This study was to characterize any potential associations between RV infection and the intestinal microbiota.

**Methods:**

Suckling mice were divided into normal group (NC) and infected group (RV) randomly. All of the suckling mice were euthanized four days post-RV infection. The virus titer was counted as fluorescent focus assay, and viral load was quantified by QPCR. Five sucking mice were randomly selected from each RV group and NC group for sample collection and pathological analysis. Mixed intestinal contents of the colon and rectum were collected from all of the suckling mice. To investigate the detailed relationship between RV infection and intestinal microbiota, the composition and distribution of intestinal microbiota from suckling mice were first analyzed using 16S rRNA sequencing technology.

**Results:**

The results of the pathological characteristics showed that vacuolar degeneration, vasodilation, hyperemia, and destruction of the intestinal epithelium were apparent in the RV group. Representative genera from Lactobacillus and Fusobacterium were enriched in the NC group, while the Enterococcus and Escherichia/Shigella genera were enriched in the RV group. Helicobacter, Alloprevotrlla, Brevundimonas, Paenibacillus, and Parabacteroides were completely undetectable in the RV group. The predicted intestinal flora metabolic function results showed that “carbohydrate metabolism” and “lipid metabolism” pathways were significantly enriched within the NC group. A significant difference has been observed in the gut microbiota composition between the two groups.

**Conclusions:**

Our results demonstrated a significant difference in the gut microbiota composition in RV-infected suckling mice as compared to the RV un-infected suckling mice group. This work may provide meaningful information regarding the bacterial genera changed during RV infection. Moreover, the changes in these bacteria may be related with the replication and pathogenesis of RV infection.

## Background

Rotavirus (RV) can infect almost all mammalian and avian species. RV is very common gastrointestinal pathogen in infants and children under 5 [1]. Despite the global introduction of vaccinations for human RV over a decade ago, RV infections still result in > 200,000 deaths annually [2–4]. The efficacy of RV vaccines may result from low standards of hygiene [5], malnutrition, and disorder of the intestinal microbiota [6, 7].

The intestinal microbiota plays an important role in host health. It has been demonstrated that the ecology and function of the microbiota are related to enteric virus infections [8] Moreover, virus infection can change the gut microbiota composition and activity. Some viruses, such as poliovirus [9], reovirus [9], norovirus [10, 11] and murine RV [12, 13], have been reported to influence the gut microbiota. Commensal bacteria have been shown to enhance the infectivity of enteric viruses through several mechanisms, such as bacterial stabilization of viral particles, help of viral adsorption target cells, and restraining of antiviral immune responses [14].

Previous studies have revealed that human RV infection in infants reduces the fecal microbiota diversity as compared to healthy infants [15, 16]. Microbiota ablation resulted in reduced RV-induced diarrhea in mice model and a more durable RV-targeted antibody response via germ-free or antibiotic approaches [17]. Another study showed that segmented filamentous bacteria (SFB) could protect mice against RV infection and associated diarrhea sufficiently [18].

*Bifidobacterium* and *Lactobacillus* species have been showed to be associated with increased extent of specific anti-RV immune responses, and subsequently, a shorter duration and severity of RV infection [19, 20]. The probiotic bacteria *Escherichia coli* Nissle and *Lactobacillus rhamnosus* strain GG have been shown to influence the binding, infectivity, and B cell immune response of human RV [21]. Additionally, antibiotics have been shown to increase the fecal output of RV, but also changes the beta diversity of gut bacterial, which further demonstrates that modification of the intestinal microbiota alters the immune response [17].

It have been shown that the gut microbiota modulates RV infection and the antibody response of the host against RV infection in animal models [8–13]. However, little studies regard alteration in the gut microbial upon RV infection. Thus, it is urgent to set up the etiological link between RV infection and the gut microbiota.

In this study, we elucidated the relationship between the gut microbiota and RV infection by characterizing the intestinal microbiota via 16S rRNA sequencing in a RV-induced diarrhea model in suckling mice. Our findings provide information regarding the development of probiotic therapy to ameliorate the symptom caused by RV infection or the identification of a microbial target that can inhibit RV replication and infection in children.

## Materials and method

### Viruses, cells, and viral load quantification

RV SA11 strain (provided by Dr Kobayashi, Osaka University, Japan) and fetal African green monkey kidney cells (MA104 cells; Cell Resource Center, IBMS, and CAMS/PUMC, Beijing, China) were used in this study. MA104 cells were grown in Dulbecco’s modified Eagle’s medium (DMEM; Thermo Fisher Biochemical, Beijing, China) supplemented with 5% calf serum (FBS; GIBCO, Paisley, UK) and 1% penicillin–streptomycin (Sigma-Aldrich, St. Louis, MO, USA) in 5% CO2 at 37 °C in a humidified incubator. RV strain SA11 was propagated in MA104 cells as in previous studies^42^. The virus titer was counted by fluorescent focus assay (FFA) in MA104 cells resulting of 10^6^FFU/mL, and viral load was quantified by QPCR [22].

Total RNA was extracted from the colorectal contents of mice by using RNAiso Plus (Takara Bio Inc, Dalian, China) following the manufacturer’s protocol. The RNA was quantified using a NanoDrop 1000 Spectrophotometer (Thermo Fisher Biochemical, Beijing, China). The cDNA was prepared from 2 μg of RNA using the following primers: forward: 5′-ATCAGCAAACTGACGAAGCG-3′; reverse: 5′-CCAACTTTTCAGCTGTCGCA-3′ (Takara Bio Inc, Dalian, China). In brief, the amplification was performed using a 10-μL volume reaction in a 96-well plate with the following conditions: 1 cycle at 94 °C for 30 s, followed by 36 cycles of 94 °C for 5 s, 60 °C for 30 s, and 72 °C for 30 s. The RV RNA copy levels were quantified by comparison with a standard curve generated using ten two-fold serial dilutions of a plasmid containing the RV *VP7* gene.

### Animal and experimental design

Twenty specific-pathogen-free female Kunming (KM) mice were provided by the Laboratory Animal Center at Jinzhou Medical University (Liaoning Province, China). All experimental groups were housed in the same specific-pathogen-free room. The living environment, feeding conditions and microbial conditions of animals in research were always consistent which was maintained on a 12-h light/dark cycle at 22 ± 2 °C with 40–70% humidity.

The female mice were paired with male mice upon delivery. The males were removed the next day, and the pregnant females were monitored daily and allowed to deliver at term. The day of birth was recorded as day 1 of life. Litter statistics and the ratio of males/females in each cage were not calculated. One lactating female mouse and her pups were maintained together in individually ventilated autoclaved cages (IVC). Suckling mice were divided into two groups: RV-infected and uninfected groups. Each group contained 10 litters of mice. There were 7–8 mice in each litter. RV-infected groups of suckling mice were orally administered 50 μL of 10^6^ PFU/mL RV strain SA-11 [12], and the uninfected group were orally administered 50 μL of phosphate-buffered saline (PBS) as a control.

All of the suckling mice were euthanized four days (the time point at which the most severe diarrhea symptoms presented) post-RV infection. Mixed intestinal contents of the colon and rectum were collected using autoclaved tweezers and stored in sterile tubes at − 80 °C. All animal experiments were performed in accordance with the Jinzhou Medical University guidelines. All of the animal experiments in this study were approved by the Animal Welfare and Ethical Review Board at Jinzhou Medical University (approval ID: 2019014). All animal infections and infectious work were performed in biosafety level 2 facilities.

### Histopathology

Five sucking mice were randomly selected from each RV group and NC group for sample collection and pathological analysis. The duodenum was harvested from the abdominal cavity immediately after the suckling mice were euthanized. For the histopathological investigation, the duodenum was fixed in a solution of 4% paraformaldehyde (0.01 M PBS, pH 7.4). For paraffin section preparation, the duodenum was dehydrated with an increasing ethanol gradient, cleared with xylene, and then embedded in wax. Three consecutive paraffin sections (5 μm thick) were used for hematoxylin and eosin (H&E) staining. For each slice, fields were randomly selected.

### DNA extraction and PCR amplification

The RV-infected and uninfected control mice groups were euthanize. Each group contained 10 litters of mice, and there were 7–8 mice in each litter. Genomic DNA was extracted from mixed intestinal contents using the QIAamp DNA Stool Mini kit (Qiagen Inc., Valencia, CA, USA) according to the manufacturer protocols. The V3–V4 region of the bacterial 16S ribosomal RNA genes (342F and 806R) was amplified by PCR using primers 341F 5′-CCTACGGGRSGCAGCAG)-3′ and 806R 5′-GGACTACVVGGGTATCTAATC-3′. The PCR protocol used in this study was as follows: 95 °C for 3 min, followed by 30 cycles at 98 °C for 20 s, 58 °C for 15 s, 72 °C for 20 s, and a final extension at 72 °C for 5 min. PCR reactions were performed in a 30-μL mixture containing 15 μL of 2× KAPA Library Amplification ReadyMix (Roche-KAPA, Shanghai, China), 1 μL of each primer (10 μM), 50 ng of template DNA, and ddH_2_O. Amplicons were extracted from 2% agarose gels and purified using the AxyPrep DNA Gel Extraction Kit (Axygen Biosciences, Union City, CA, USA) according to the manufacturer’s instructions. The resulting library was analyzed with a Thermo NanoDrop 2000 spectrophotometer (ThermoFisher, Shanghai, China) and 2% agarose gel electrophoresis. Once the library passed the quality inspection, it was quantified by Qubit and mixed according to the data requirements of the quantification and normalization of individual PCR products.

### Sequencing of 16S rRNA gene amplicons

The PCR products were quantified using Qubit (Invitrogen, Carlsbad, CA, USA), multiplexed at an even concentration, and subjected to 400–450 bp pair-end sequencing. The adaptor was added to the products, and the samples were sequenced on an Illumina MiSeq platform (Illumina, Inc., San Diego, CA, USA). The reads were assembled and used for subsequent 16S analysis. The assembled reads were filtered to acquire clean reads. Reads with an average quality value below 20 and number N that was more than 3 was removed, then the sequences spanning the entire V3–V4 amplicon were matched using PANDAseq [23]. Merged sequences with 97% nucleotide sequence identity (97% identity) were binned into operational taxonomic units (OTUs) using UPARSE [24]. Based on the RDP classifier, a representative sequence of each OTU was assigned to a taxonomic level in the RDP database using 0.8 as the minimum confidence threshold [25].

### Statistical analysis of the data

Alpha diversity indices, including the number of OTUs, observed species diversity, and Shannon and Simpson indices, were calculated by normalizing the number of clean reads in all samples to 47,504 sequences using mother software [26]. Rarefaction curves were analyzed with mothur and plotted using R. A representative sequence was chosen from each OTU by selecting the sequence that had the largest number of hits in the OTUs. The non-parametric Wilcoxon test (Wilcox. Test in R) was performed for each index of alpha diversity. Rank sum test was used to screen the alpha diversity indices with significant differences under different conditions.

Beta diversity was calculated for the normalized OTU table using UniFrac distance matrices [27, 28] in order to determine the amount of bacterial diversity shared between the two groups. Principal coordinate analysis (PCoA) of bacterial communities was performed using weighted UniFrac distances based on the presence and absence of OTUs, and the plot was generated using PERMANOVA, which was also performed using weighted UniFrac distance to test for differences in bacterial community composition in the samples from the two groups.

Linear discriminant analysis effect size (LEfSe) analysis was used to determine the features most likely to explain differences between the RV-infected group and healthy control group. Different features with an LDA score were identified [29]. The *p* value was set at *p* < 0.05, and the threshold on the logarithmic linear discriminant analysis (LDA) score was 2 [30]. In addition, heatmap analysis was performed to compare the significant differences at the genus level in the RV-infected and uninfected groups. PICRUSt (version 1.0.0, http://picrust.github.com/) [31] was performed to predict the microbial community function of the two groups. Finally, a statistical analysis of the taxonomic and functional profiles (STAMP, version 2.1.3, http://kiwi.cs.dal.ca/Software/STAMP) [32] was used for further exploration in level 2 of the KEGG analysis using Reconstruction of Unobserved States (PICRUSt) analysis.

## Results

### Viral infection and histopathology

**Fig. 1:**
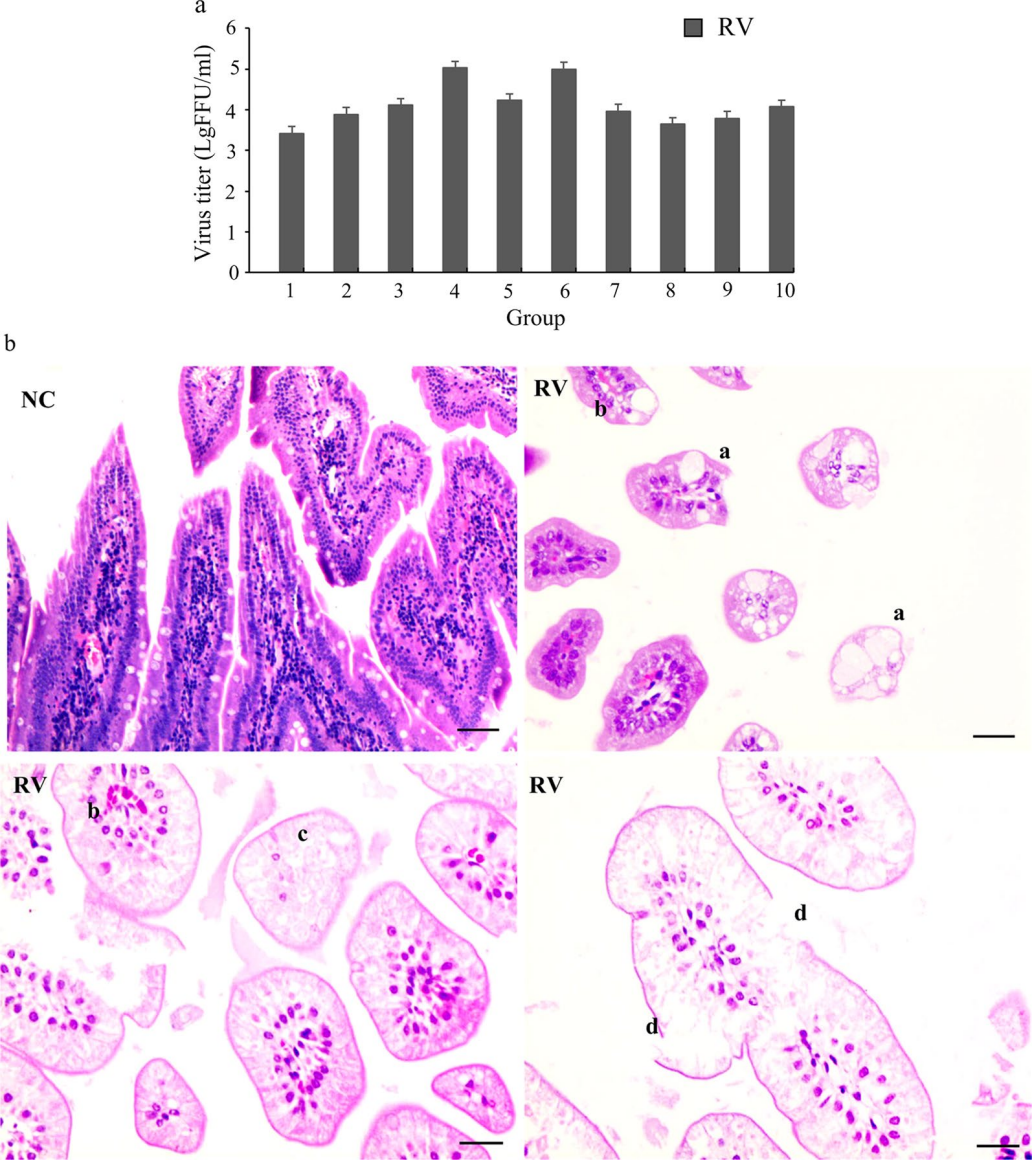
Changes in suckling mice infected with rotavirus strain SA-11. **a** The viral load in the feces of the suckling mice in the RV group was quantified by a fluorescent focus assay (FFA) with MA104 cells. No virus was detected in NC group. The RV group contained 10 litters of mice, and there were 7–8 mice in each litter. Each experiment was repeated three times. **b** Histopathological changes within the duodenum of mice challenged with RV-SA11 as demonstrated by H&E staining. Five sucking mice were randomly selected from each RV group and NC group for sample collection and pathological analysis. a: vacuolar degeneration of intestinal epithelial cells; b: congestion and edema of the stroma, dilation, and congestion of blood vessels distributed within the villi of the small intestine; c: necrosis of cells; and d: destruction of the intestinal epithelium. Scale bar = 50 μm (a); 20 μm (b, c, and d)

All RV-infected suckling mice in the RV group had watery feces after infected 4 days. These clinical sign were not shown in the suckling mice in the normal control (NC) group. Quantification of the viral titer via fluorescent focus assay (FFA) showed values ranging from 3 × 10^5^ to 5.2 × 10^7^ PFU/mL. No virus was detected in the NC group (Fig. 1a). Five sucking mice were randomly selected from each RV and NC group for sample collection and pathological analysis. About 90% of the samples showed above reported phenomenon. The pathological characteristics of the duodenum showed vacuolar degeneration of the intestinal epithelial cells and congestion and edema of the stroma distributing within the villi of the small intestine in the RV group (Fig. 1b). There was no pathological damage in the NC group.

### The microbial diversity in the colorectal contents of suckling mice infected with RV was decreased

**Fig. 2:**
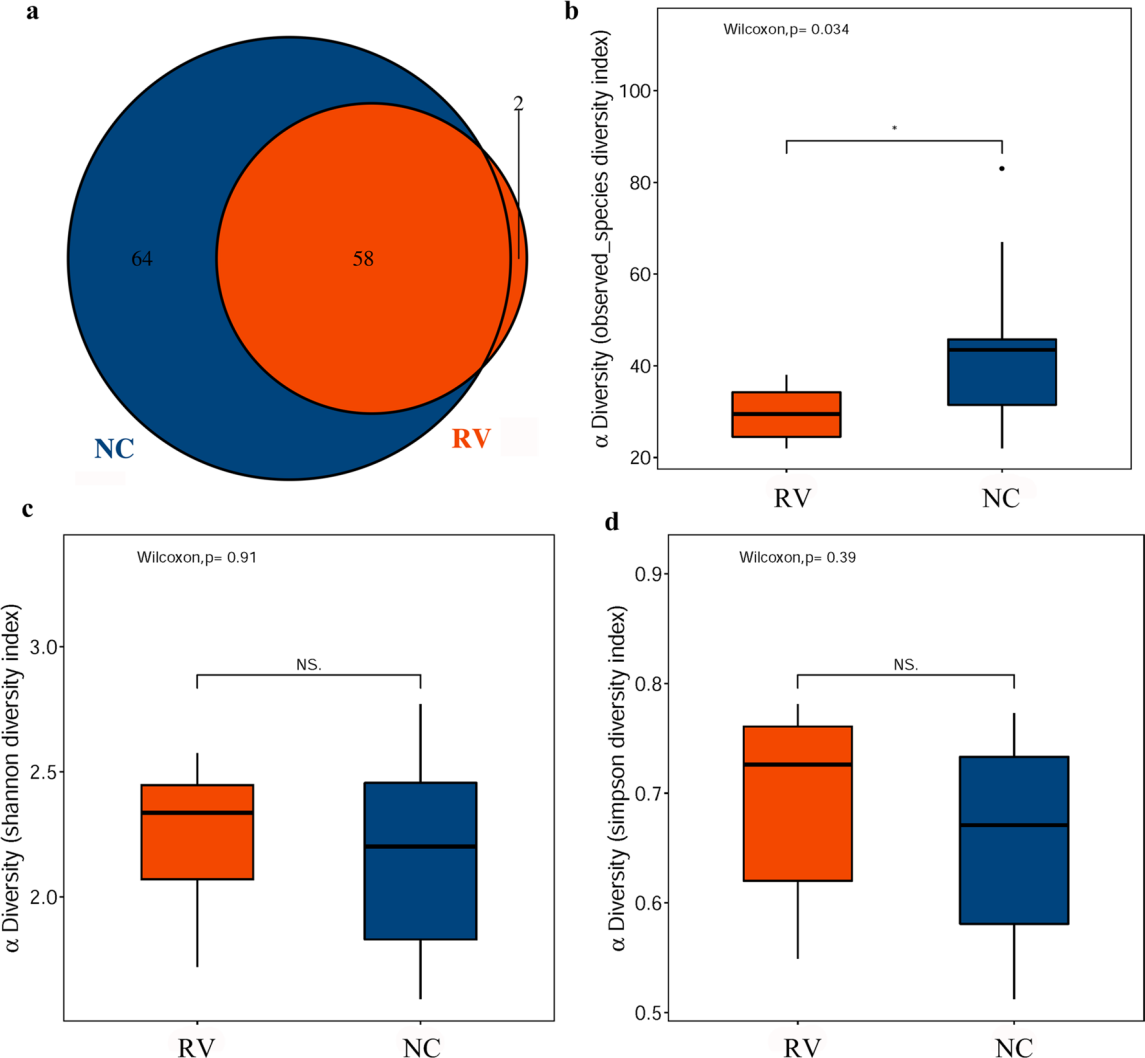
The composition and α diversity of the gut microbiota in the normal control (NC) and RV-infected (RV) suckling mice were detected by 16S rRNA sequencing. The RV and NC groups contained 10 litters of mice. There were 7–8 mice in each litter. **a** A Venn diagram showing the overlap in the differential abundance of the operational taxonomic units (OTUs) in the NC and RV groups. **b** The bacterial diversity in the RV and NC groups was estimated by the observed-species diversity index. **c** The bacterial diversity in the RV and NC groups was estimated by the Shannon index. **d** The bacterial diversity in the RV and NC groups was estimated by the Simpson index

After runs on an Illumina MiSeq PE250 platform and quality filtering as described in the methods, a total of 1,182,607 merged sequences produced from all samples result in an average yield of 59,130 ± 2571 sequences/sample. According to alpha diversity analysis, which takes into account both sequencing saturation and sample integrity, 47,504 reads were randomly selected for each sample. The sample sequence diversity and richness were assessed on account of the operational taxonomic unit (OTU) counts in every sample as shown in Table 1. A Venn diagram showed that there were 58 OTU sequences in the RV groups and 64 OTU sequences in the NC group, with 58 OTUs shared (Fig. 2a). The number of OTUs in the NC group was significantly higher than that in the RV group, manifesting a significant difference in the number of OTUs between the two groups (Wilcox, *p* = 0.034) (Fig. 2b). However, There were no significant difference in Shannon and Simpson indices between the RV and NC groups within the experiment (Wilcox, *p* = 0.91 and *p* = 0.39, respectively) (Fig. 2c–d).

**Table 1:** Number of OTUs per group and estimate of sequence diversity and richness

Sample name	OTUs	Reads	observed_species	Shannon	Simpson	Goods_coverage
RV-1	35	62,257	35	2.066	0.706	0.999
RV-2	25	60,752	23	2.476	0.768	0.999
RV-3	27	64,632	26	2.305	0.731	0.999
RV-4	23	60,228	22	1.741	0.586	0.999
RV-5	28	60,919	28	2.081	0.591	0.999
RV-6	25	58,502	24	1.719	0.549	0.999
RV-7	39	61,331	38	2.467	0.765	0.999
RV-8	32	61,631	32	2.575	0.781	0.999
RV-9	37	55,827	37	2.388	0.721	0.999
RV-10	32	58,004	31	2.368	0.748	0.999
NC-1	27	55,601	26	1.619	0.552	0.999
NC-2	24	58,633	22	2.404	0.752	0.999
NC-3	47	56,388	46	2.632	0.773	0.999
NC-4	44	60,228	43	2.021	0.669	0.999
NC-5	69	60,919	67	2.473	0.732	0.999
NC-6	46	57,250	45	1.765	0.536	0.999
NC-7	44	57,272	44	1.590	0.512	0.999
NC-8	83	56,172	83	2.770	0.667	0.999
NC-9	42	60,303	42	2.318	0.734	0.999
NC-10	28	58,004	28	2.084	0.673	0.999

### RV infection altered the microbiota composition in the colorectal contents of suckling mice

**Fig. 3:**
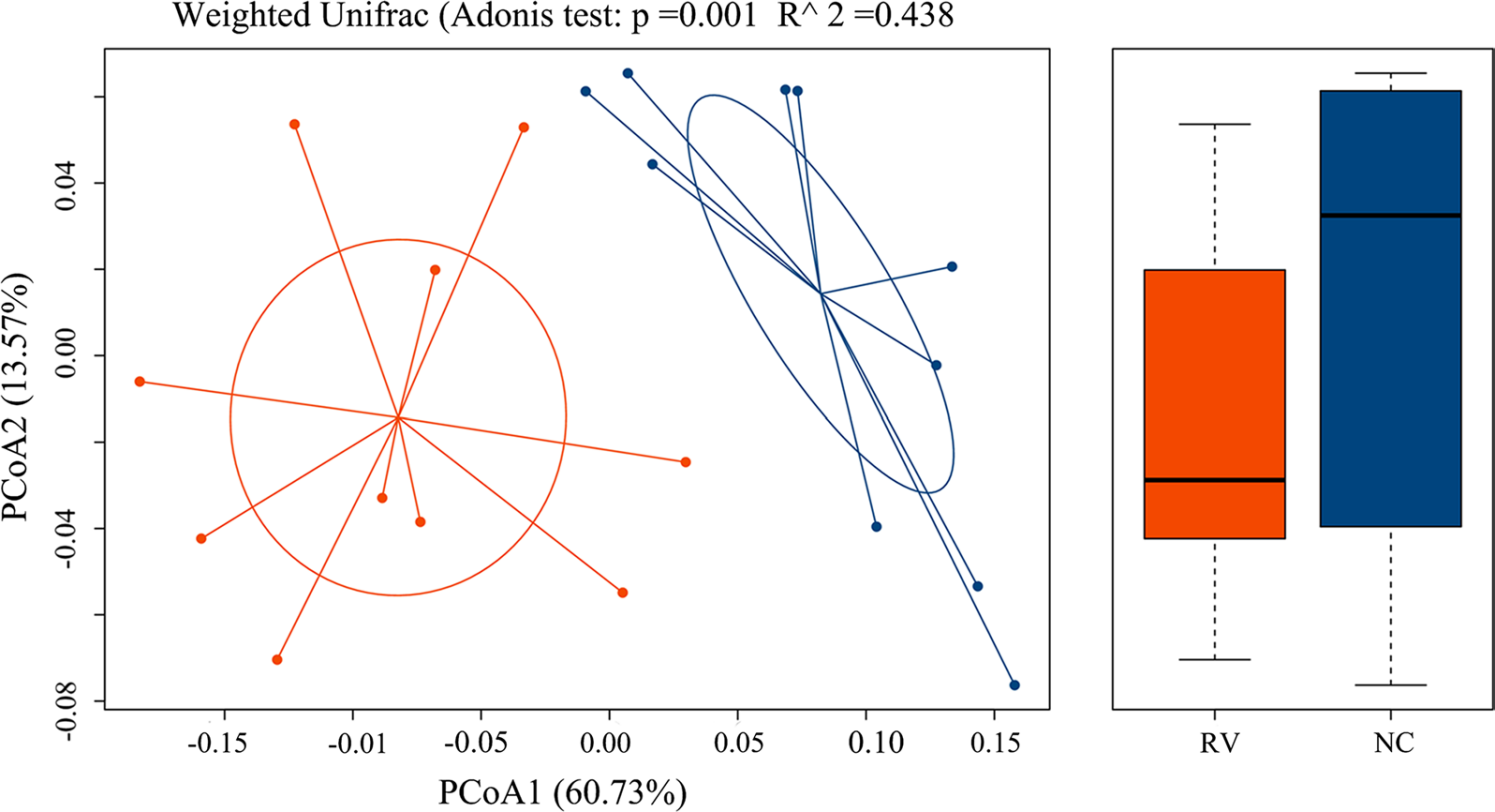
Exploration of the beta diversity in the normal control (NC) and RV-infected (RV) groups was assessed by Weighted Unifrac ANOSIM analysis. A principal coordinate analysis (PCoA) plot of the similarities between the different groups based on UniFrac distance. Principal components PCOA1 and PCOA2 explained 60.73% and 13.57% of the variance, respectively. Analysis of Adonis of the bacterial communities in the colorectal contents of the RV and NC groups was based on unifrac distance (R < 0.438 > 0 indicates that the differences between the groups are significant; *p* < 0.001 indicates that the differences are significant)

In order to contrast the differences in the bacterial community composition between the RV and NC groups, the beta diversity in both groups were calculated. We utilized principal coordinate analysis (PCoA) of variance (PERMANOVA) (R vegan package function Adonis) analyses to visualize the differences in the microbial community structures. Figure 3 is a weighted UniFrac PCoA on account of comparison of the microbial community from the colorectal contents of both groups (Adonis test *p* = 0.001, R^2^ = 0.438). These results showed that the RV and NC groups had a clear separation and distinct differences in the bacterial composition between the RV and NC groups.

### Species classification and relative abundance analysis

**Fig. 4:**
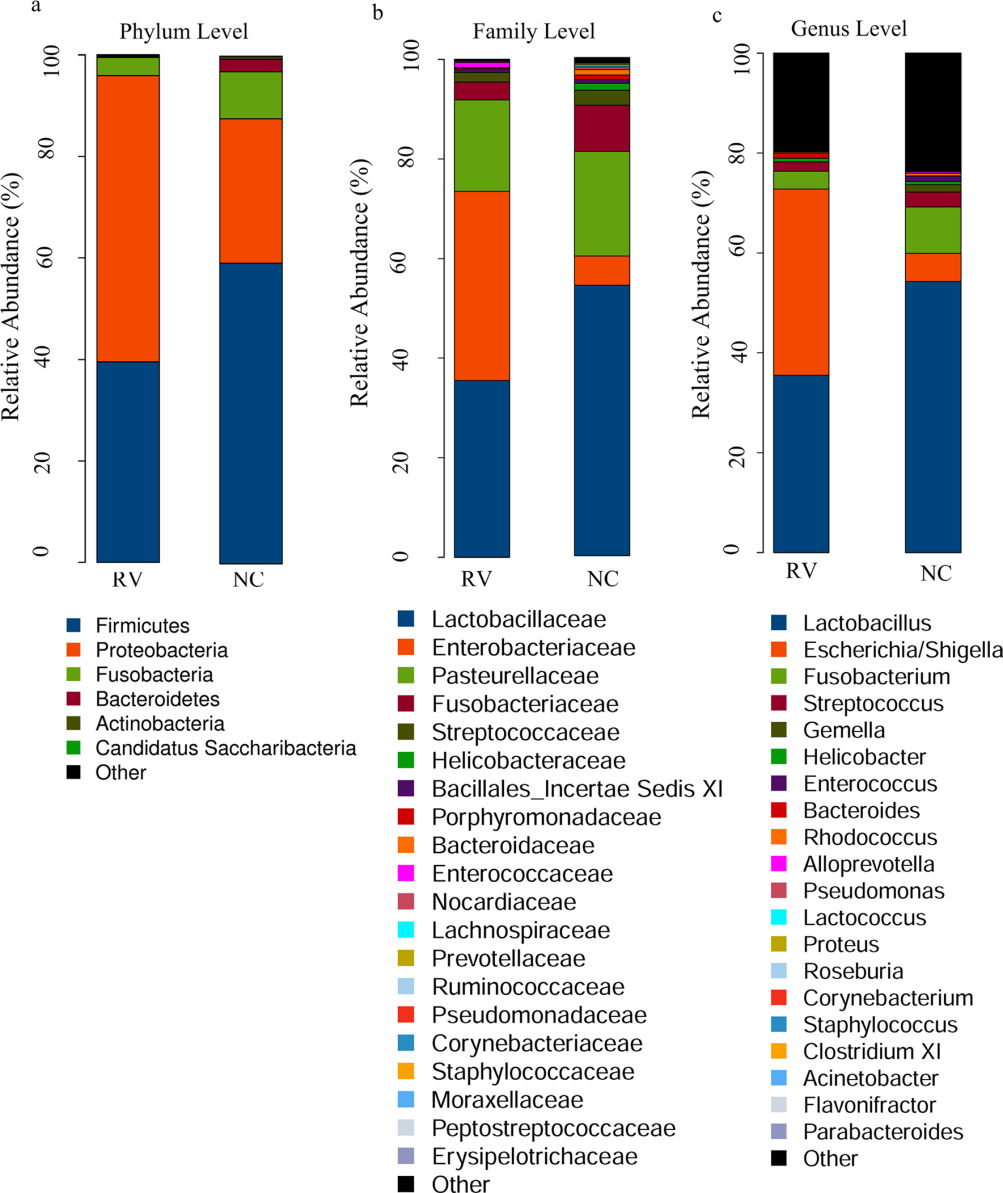
Aggregate microbiota composition at different taxon levels in normal control (NC) and RV-infected (RV) mice. A bar plot of the identified bacterial phyla in the analyzed samples. The abundance of bacteria is shown at the phylum (**a**), family (**b**) and genus (**c**) levels. The horizontal coordinate is the RV and NC groups. The vertical coordinate is the relative abundance of the species. The different colors correspond to the different species, and the length of the color block represents the relative abundance of the species

According to the results of species annotation, the abundance within samples was analyzed at the phylum, class, order, family, and genus levels. An examination of the forecasted taxonomic profiles at the phylum level for all the samples showed that *Proteobacteria* (56.40%) was the major phylum within the mixed colorectal contents of the RV group, exceeding both *Firmicutes* (39.55%) and *Fusobacteria* (3.58%) (Fig. 4a). An examination of the forecasted taxonomic profiles at the family level for all of the samples showed that *Enterobacteriaceae* (38.12%) was the main family in the mixed intestinal contents of the RV group, exceeding *Lactobacillaceae* (35.57%) and *Pasteurellaceae* (18.44%) (Fig. 4b). Notably, at the genus level, there was a decrease in the abundance of both *Lactobacillus* (70.57 vs.46.29 percentage) and *Helicobacter* (1.89 vs. 0%) in the RV group as compared to that in the NC group. Additionally, the abundance of *Escherichia/Shigella* (7.40 vs. 43.48%) and a decrease in the abundance of *Fusobacterium* (12.43 vs. 4.74%) in the RV group as contrasted to the NC group (Fig. 4c).

### A significant differences in the taxonomical composition between the RV and NC groups

**Fig. 5:**
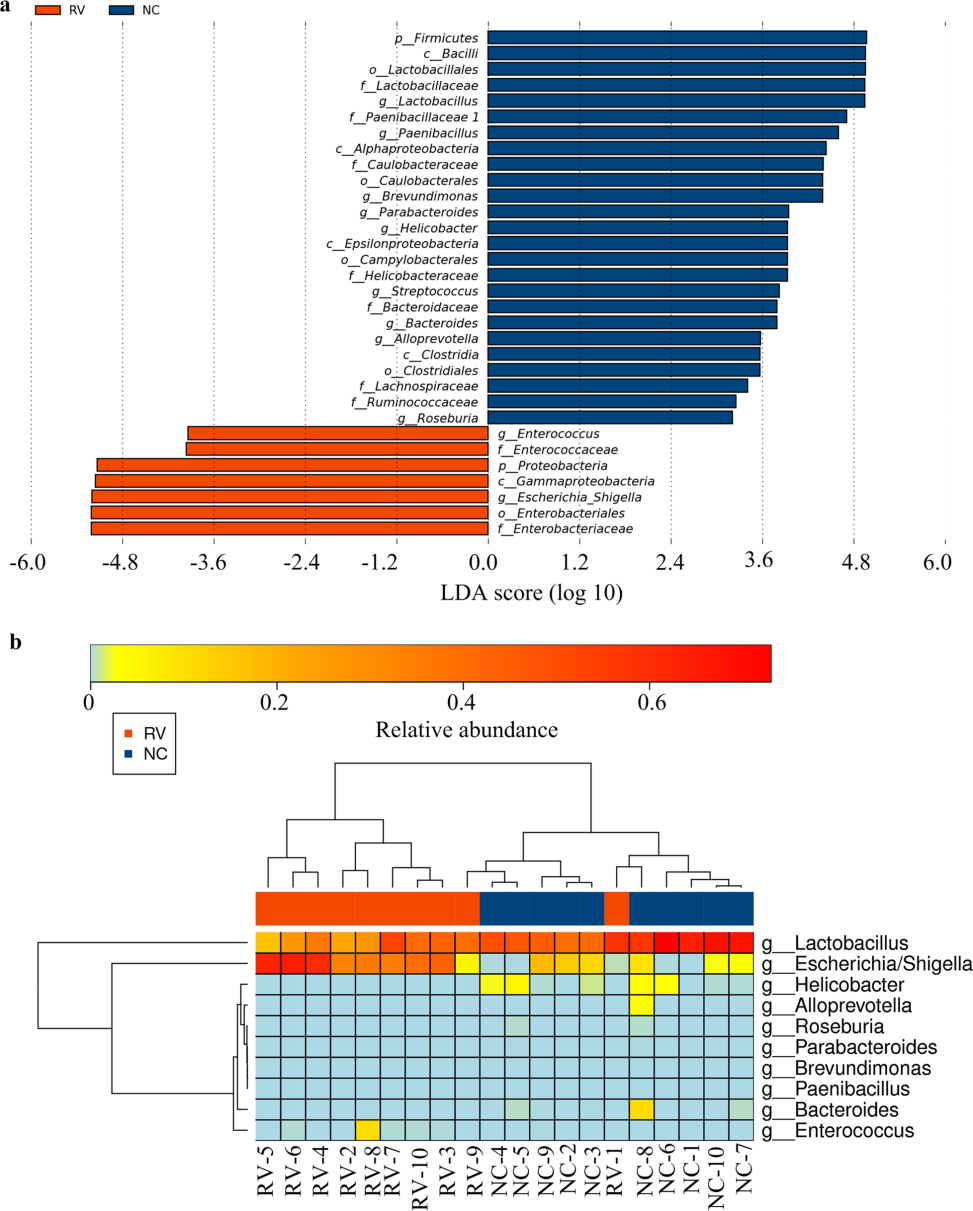
Different structure of the microbiota in the colorectal contents of the normal control (NC) and RV-infected (RV) groups. **a** Taxonomic biomarkers found in the RV (red) and control (blue) groups by linear discriminant analysis effect size (LEfSE). Statistical analysis of the LDA scores obtained from the microbial groups with significant differences in the RV and NC groups through regression analysis (LDA threshold 2). **b** Heatmap of the significant differences at the genus level in the RV and NC groups

Linear discriminant analysis effect size (LEfSe) analysis was carried out to identify the salient features of the two groups. *Firmicutes* was the predominant phylum in the NC group, while the enriched phylum in the RV group was *Proteobacteria*. Notably, at the genus level, *Lactobacillus, Paenibacillus, Brevundimonas, Parabacteroides, Bacteroides,* and *Alloprevotella* were the predominant genera in the NC group, while the enriched genera in the RV group included both *Enterococcus and Escherichia/Shigella* (Fig. 5a). The distinctive features between the two groups at the genus level are listed in Table 2. All species at the genus level were analyzed. The species that failed to pass the *p* value screening were not shown. There were 10 genera with obvious differences between the RV and NC groups that were identified via a Wilcoxon rank sum test. Significant differences at the genus level between the RV and NC groups were illustrated by a heatmap (Fig. 5b).

**Table 2:** Distinctive features at the genus level between the two groups

Taxon name	Mean (NC)	Mean (RV)	*p* Value	fdr
g__Alloprevotella	0.003214466	0	0.034983096	0.136434
g__Bacteroides	0.01103907	4.84E−05	0.025905743	0.12629
g__Brevundimonas	3.37E−05	0	0.014866251	0.096631
g__Enterococcus	0.000101044	0.01064963	0.020251648	0.112831
g__Escherichia/Shigella	0.056471034	0.372987538	0.001504687	0.029341
g__Helicobacter	0.014278798	0	0.000751179	0.029296
g__Lactobacillus	0.542832183	0.354740653	0.006841456	0.088939
g__Paenibacillus	2.32E−05	0	0.034983096	0.136434
g__Parabacteroides	0.000109464	0	0.014866251	0.096631
g__Roseburia	0.000618895	6.32E−06	0.014557687	0.096631

### Predicted metagenomic functions that occur during RV infection

**Fig. 6:**
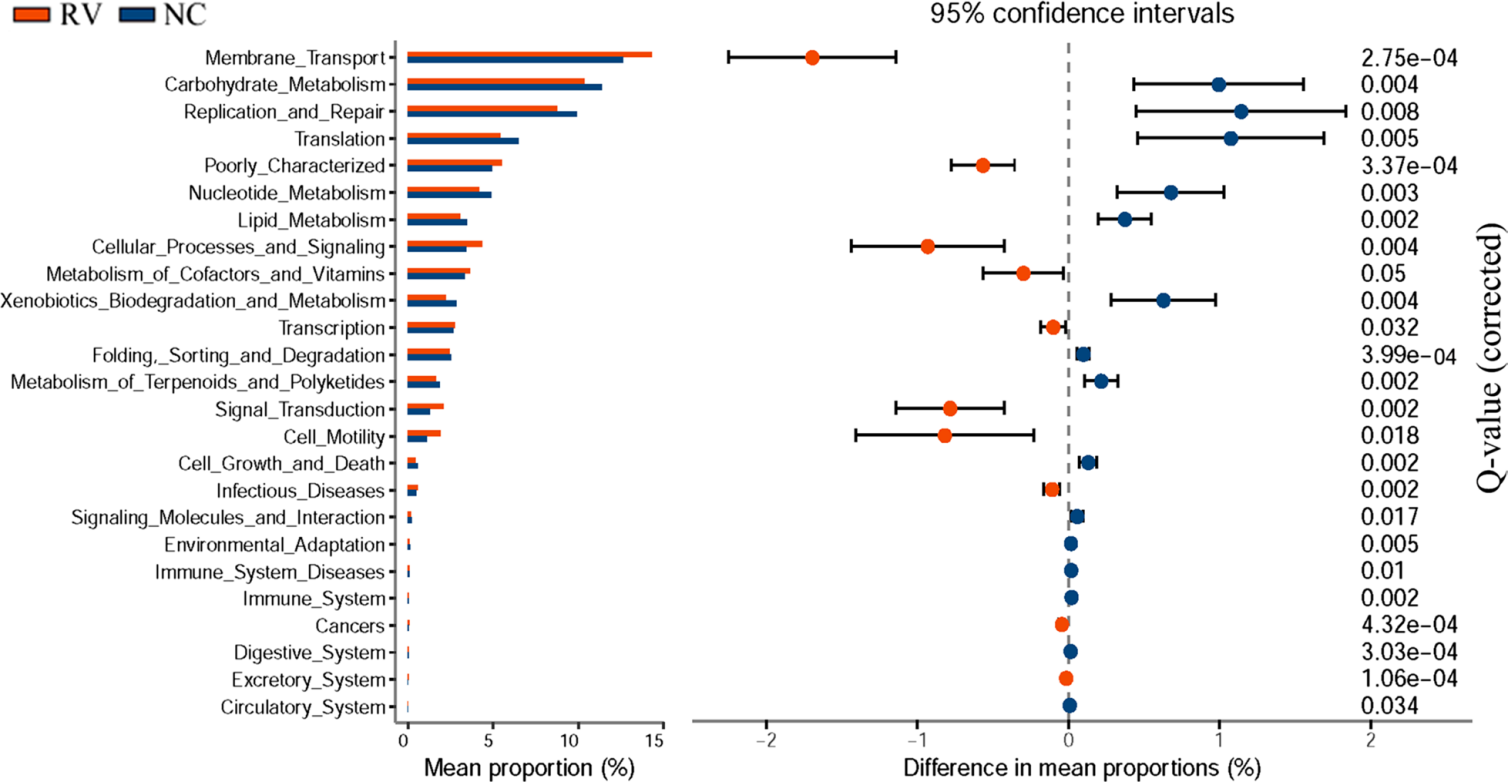
Significant KEGG pathways in the fecal microbiome of RV-infected and NC control at level 2 as identified by STAMP software. In STAMP, differences in the abundances between the RV and NC groups were compared using a White’s non-parametric t test. Confidence intervals were estimated using a percentile bootstrapping method (10,000 replications). NC group (blue); RV-infected group (red); Only comparisons with a *p* < 0.01 are shown

In order to gain insight into the differences between the microbiota functions between the RV and NC groups, Phylogenetic Investigation of Communities by Reconstruction of Unobserved States (PICRUSt) was used to forecast the potential metagenomes from the community profiles of the normalized 16S rRNA genes. LEfSe was used to analyze the influence of metabolic pathways identified via the Kyoto Encyclopedia of Genes and Genomes (KEGG, http://www.genome.jp/kegg/) database and calculate the significant differences between the two groups. The results showed that there were 10 pathways from level 2 that were enriched in the RV group (*p* < 0.001, White’s non-parametric t test) and 13 pathways that were enriched in the NC group. STAMP (Statistical Analysis of Metagenomic Profiles) analysis was used to predict the differences of the metabolic pathways of KEGG. The results showed that there were 9 pathways enriched in the NC group: (1) carbohydrate metabolism, (2) replication and repair, (3) translation, (4) nucleotide metabolism, (5) lipid metabolism, (6) xenobiotics biodegradation and metabolism, (7) folding, sorting, and degradation, (8) metabolism of terpenoids and polyketides, and 9) cell growth and death. Eight pathways were enriched in the RV group: (1) membrane transport, (2) poorly characterized, (3) cellular processes and signaling, (4) metabolism of cofactors and vitamins, (5) transcription, (6) signal transduction, (7) cell motility, and (8) infectious diseases (Fig. 6). In short, these data indicated that RV infection changes the metabolic functions of the intestinal microbiota in suckling mice. The metabolic functions identified here need further investigation in order to understand the role they in RV infection.

## Discussion

Enteric viral infections remain a major public health challenge. Since enteric viruses first encounter host cells amidst the microbiota, the microbiota composition might influence RV infection [18]. Whereas, only narrowed amount of information regarding the correlation between the gut microbiota composition and RV infection. Therefore, the effects of RV infection on the intestine and intestinal microbiome in suckling mice were investigated in this report.

A previous report showed that gut microbial diversity was cut down in infants infected with human RV, however the enrichment of diversity was observed in healthy children’s, such as phylum Firmicutes was abundant [15]. Additionally, it has been shown that there is a reduction of microbial diversity in children with RV infection as compared to children without infection [33, 34]. In this work, we also observed low bacterial diversity in the RV group as compared to healthy mice. Therefore, the gut microbiota appears to take an important role in diarrhea-associated processes. The intestinal symbiotic role in regulating viral infections has been realized. An RV-infected suckling rat model was formerly taken to assess the effects of prebiotic mixtures in fermented milk on the reforming the course of infectious and amendment the immune response [13]. The gut microbiota has also been shown to affect intestinal virus replication in mice [9]. These study demonstrated that as RV infection model, suckling mice was used to assess the change of intestinal flora is feasible.

Higher diversity and integrity of intestinal flora are beneficial to intestinal ecosystem [35]. The integrity of the microbiota for more and more physiological process is indispensable. The breakdown of homeostasis of the microbiota is associated with a variety of pathological states [35]. Consequently, interference of the microbial balance (dysbiosis) may have substantial consequences for the metabolism and adaptive immune responses of the host. Importantly, commensal microbiota protect against invading bacterial pathogens. Loss of intestinal flora diversity is the most common symptom of intestinal disorders. Restoration of the intestinal flora diversity may be an option for the treatment of people with risk [36]. In order to discuss the influence of RV infection on the relative abundance of specific microbiome taxa, we identified the bacterial genera that were different between RV-infected suckling mice and healthy suckling mice. We found alterations in the gut microbiota of the RV group. The relative abundance of *Proteobacteria* and *Candidatus saccharibacteria* was increased, while the abundance of *Firmicutes*, *Fusobacteria*, *Bacteroidetes,* and *Actinobacteria* was decreased as compared to NC group, which is consistent with a previous report where the phylum *Bacteroidetes* was reduced in RV-induced diarrheal disease children [37]. The gut microbiota in healthy mice was dominated by *Firmicutes* (59.29%), followed by *Proteobacteria* (28.43%), and *Fusobacterial* (9.29%) during the neonatal period.

Notably, there was significant increase in the genera *Escherichia/shigella* in RV-infected mice. However, *Lactobacillus*, *Fusobacteria*, *Streptococcus,* and *Helicobacter* were decreased. *Lactobacillus* was also found decreased in a BALB/c model with rhesus rotavirus (RRV) infection [38]. The present LEfSe analysis showed that *Helicobacter*, *Alloprevotrlla*, *Brevundimonas*, *Paenibacillus*, and *Parabacteroides* were completely undetectable in the RV-infected group. Former studies have shown that *Lactobacillus* and *Helicobacter* are regarded as probiotics with certain protective effects, which can eliminate infections, attenuating both GI diseases and produce lactate and butyrate [39]. Probiotics have been increasingly used to enhance the oral vaccine reactions and treat some intestinal infection and inflammatory of GI in children [40]. In probiotics, gram–positive (G+) probiotics, such as *Lactobacillus* spp. or *Bifidobacteria* spp., have been used in randomized clinical trials in humans [41, 42] and experimental studies [19, 20, 43, 44] to reduce the severity of the diarrhea caused by RV. *Lactobacillus rhamnosus* GG (LGG) has been extensively investigated for its beneficial health effects, such as shortening the duration of HRV diarrhea and enhancing HRV-specific immune responses in children [41, 45]. However, one study regarding *Lactobacillus* probiotics showed that probiotic supplementation in Indian infants did not substantially boost RV vaccine response in a randomized controlled trial [46]. Therefore, the effect of probiotics use for diarrhea is still unclear. A modest effect of probiotics supplementation deserves further investigation.

In this study, we observed a significant increase in the *Escherichia/shigella* genera in the RV-infected group, suggesting that RV likely promotes the disruption of the epithelial integrity and further induces changes in the abundance of *Shigella*. Although this study suggests that E. coli may have potentially detrimental effects on the intestine. However, some reports show that E. coli Nissile has been shown to ameliorate diarrhea in RV infection in neonatal gnotobiotic (Gn) piglets models. These studies highlight strain specificity in the Escherichia may play a role in rotavirus infection [47–49].

The abnormal abundance of *Escherichia*/*Shigella* in the intestine may be closely associated with many diseases. *Some studies have shown that an increase in intestinal Shigella in patients with rheumatoid arthritis* [50] *and compensated heart failure* [51] *is accompanied by a significant decrease in Lactobacillus and Firmicutes.* In the gut microbiota of mice infected with Shigella, Lactobacillus supplementation inhibited the proliferation of *Shigella* within the intestinal flora of mice [52]. In this study, these same changes in the bacterial flora were observed in the intestine of suckling mice infected with RV virus. These results showed that changes in the abundance of *Lactobacillus*, *Firmicutes*, and *Escherichia*/*Shigella* correlated in intestinal homeostasis.

In the present study, we predicted the unobserved character states in the bacterial community by using PICRUSt, which is generally applied to investigate the intestinal function of suckling mice. With respect to the results of the LEfSe and STAMP analyses, “carbohydrate metabolism” and “lipid metabolism” pathways were significantly enriched in the NC group. Multiple studies have suggested that the gut microbiota influences host metabolism and function [53]. Recent research has identified many virus-specific metabolic pathways and that eukaryotic viruses induce mass changes of metabolism of the host [54]. Lipid plays an important role in viral infection because lipids are structural elements of cells and viral membranes [55], membrane fusion, envelopment, remodeling, and lipid compounds, such as cholesterol and sphingolipids, are vital for viral replication. Viruses also make lipids at sites of replication by promoting lipid biosynthesis [56]. It has been reported that hepatitis C [57] and poliovirus [9] influence lipid metabolism and bind specific microbe-associated surface polysaccharides, enhancing viral attachment to host cells.

Virally-mediated manipulation of lipid metabolism is important for viral infection, and viruses have evolved multiple mechanisms to ensure that the host-cell lipid metabolism is successfully hijacked to support viral infection. A previous study reported that RV replication was susceptible to inhibitors targeting various lipid synthetic enzymes [58]. According to our data, “carbohydrate metabolism” and “lipid metabolism” pathways are an important part of the host metabolism, which is potentially influenced by the response of the intestinal microbiota to RV infection.

## Conclusion

The gut microbiota plays an essential role in RV infection processes. Our results demonstrated a significant difference in the gut microbiota composition in RV-infected suckling mice as compared to the RV un-infected suckling mice group. This research provides meaningful information regarding the bacterial genera that changed during RV infection. However, there are some limitations in this study. The simian rotavirus strain SA11 was used in this experiments. Although the SA11 strain has been widely used in murine models, homologous mouse EDIM strain for intestinal flora analysis in murine models should be used for further study. Probably, more accurate information could be obtained by the enlargement of the mouse number and multiple sampling timepoint. In the future, we will further explore the important role of different bacterial genera of intestinal microbiota in rotavirus replication and study these effect of bacteria on the replication of RV and RV infection.

## Data Availability

The datasets generated for this study are available in the Genbank and Sequence Read Archive (SRA) of the National Center for Biotechnology Information (NCBI) database, PRJNA701531.

## Abbreviations

RV: Rotavirus
SFB: Segmented filamentous bacteria
KM mice: Kunming mice
SPF: Specific-pathogen-free female
PBS: Phosphate-buffered saline
OTUs: Operational taxonomic units
LDA: Logarithmic linear discriminant analysis
STAMP: A statistical analysis of the taxonomic and functional profiles
FFA: Fluorescent focus assay
NCgroup: The normal control group
RVgroup: RV-infected group
PCoA: Principal coordinate analysis
IVC: Individually ventilated autoclaved cages
LEfSe: Linear discriminant analysis effect size
PICRUSt: Reconstruction of unobserved states
LEfSe: Linear discriminant analysis effect size
STAMP: Statistical analysis of metagenomic profiles
